# Axonal Transport Failure as a Cellular Mechanism of Diabetic Neuropathy

**DOI:** 10.3390/cells15121078

**Published:** 2026-06-14

**Authors:** Bernard Kordas, Judyta K. Juranek

**Affiliations:** Department of Human Physiology and Pathophysiology, School of Medicine, Collegium Medicum, University of Warmia and Mazury, 10-082 Olsztyn, Poland

**Keywords:** diabetic neuropathy, axonal transport, axonal maintenance, cytoskeleton, RAGE, DIAPH1, carbonyl stress, glycation, mitochondrial dysfunction, neurodegeneration

## Abstract

**Highlights:**

**What are the main findings?**

- Diabetic neuropathy reflects convergent structural and functional injury to long axons. It is accompanied by glial, immune, and vascular dysfunction.
- Carbonyl stress and AGEs damage the axonal cytoskeleton and may contribute to transport vulnerability through mechanisms that include RAGE–DIAPH1 signaling.

**What are the implications of the main findings?**

- Future therapies should protect axon integrity and transport, and not only target relief of symptoms.
- Future studies should pair molecular mechanisms with structural and functional nerve readouts.

**Abstract:**

Diabetic neuropathy is typically diagnosed with distal sensory and nerve conduction abnormalities. These symptoms may reflect earlier disturbances of axonal maintenance. This review examines axonal transport and cytoskeletal failure as convergent cellular mechanisms of diabetic axonopathy. Long peripheral axons are particularly vulnerable to damage because their integrity depends on continuous communication between the neuronal soma and distal terminals. This process involves the continuous renewal of cytoskeletal and functional proteins and the involvement of organelles such as mitochondria. Diabetes in experimental models disrupts this system at several levels. It slows cargo transport. The supply of neurofilaments, tubulin and retrograde signaling is reduced, and regenerative growth after injury is weakened. Carbonyl stress and AGEs cause modifications of neural proteins, the extracellular matrix, vascular barriers, and the excitability of sensory neurons. RAGE ligands, including AGEs and the proteins HMGB1 and S100, link the diabetic tissue environment to redox and inflammatory signaling. This occurs in neural and glial compartments, as well as in vascular tissue and the immune system. RAGE interacts with DIAPH1 to activate GTPase signaling and remodel the cytoskeleton. The RAGE–DIAPH1 interaction provides a plausible route from diabetic ligand accumulation to cytoskeletal remodeling. These observations provide a mechanistic context for axonal transport, although not all represent direct measurements of cargo movement. Direct evidence for transport impairment comes mainly from experimental studies showing altered slow cytoskeletal transport, impaired retrograde signaling, and weakened regenerative responses. This work highlights the possibility of developing therapies that go beyond symptomatic relief. Verifying the effectiveness of interventions in protecting axonal transport and nerve fiber integrity in diabetic neuropathy may be therapeutically beneficial.

## 1. Introduction

Diabetic neuropathy (DN) develops as a progressive disturbance of neural maintenance before terminal axonal loss becomes clinically obvious. Distal symmetric diabetic sensorimotor polyneuropathy is classically defined as a chronic, symmetrical sensorimotor polyneuropathy that usually follows a pattern that depends on fiber length, with the distal portions of long peripheral axons being especially vulnerable to chronic hyperglycemia and related metabolic derangements [1]. This definition captures the clinical pattern but does not explain why distal axonal compartments are affected so early. A mechanistic explanation begins with axonal length. Peripheral sensory and autonomic axons depend on continuous exchange between the neuronal soma and distal terminals. Cytoskeletal proteins, mitochondria, vesicles and ion channels, enzymes, membrane precursors, and trophic factors must move across distances that are unusually large at the cellular scale [2]. This places axonal transport among the early cellular processes that may be compromised during diabetic nerve injury, rather than a late-stage indicator of degeneration.

Experimental work has long supported this interpretation. Diabetes impairs the slow axonal transport of neurofilament proteins, tubulin, actin, and glycolytic enzymes in peripheral nerves [3]. Mutant diabetic mice exhibit altered axonal transport of these proteins, suggesting that the supply of structural cargoes is disrupted across models of diabetes [4]. Rats with diabetes induced by streptozotocin (STZ) show impaired slow axonal transport of cytoskeletal proteins in the sciatic nerve (SN) after injection of a radiolabeled precursor into the lumbar ganglia and spinal cord [5]. In chronic diabetes, reduced myelinated fiber size correlates with loss of axonal neurofilaments, linking impaired cytoskeletal supply to axonal atrophy [6]. In rats with long-term diabetes induced by streptozotocin (STZ), neurofilament and α-tubulin mRNA expression is reduced in L4–L6 dorsal root ganglia, with parallel loss of neurofilaments and microtubules in distal sensory axons [7]. Moreover, type 1 diabetic BB/Wor rats show a weakened L4–L5 dorsal root ganglia (DRG) cytoskeletal response after SN crush, involving neurofilament and β-tubulin, with affected axonal elongation and restoration of fibers [8]. The literature provides extensive evidence of transport defects in slow cytoskeletal cargoes and retrograde signaling. For several upstream pathways, the evidence is best interpreted as a mechanistic context that may explain why transport failure develops. Accordingly, the review distinguishes between direct evidence of altered axonal transport and evidence for mechanisms that may compromise transport competence. Direct evidence includes altered slow transport of cytoskeletal proteins, impaired retrograde transport, and defective regenerative cytoskeletal responses. Mechanistic evidence includes mitochondrial dysfunction, carbonyl stress, neuroimmune activation, Schwann-cell injury, vascular dysfunction, and cytoskeletal remodeling when these processes can plausibly affect ATP supply, cytoskeletal tracks, cargo handling, or axon-to-soma communication.

Although direct demonstrations of impaired axonal transport in diabetic nerves come from classical radiolabeling studies, newer works reframe transport deficits in terms of mitochondrial dynamics and immune signaling. Because direct transport data are limited, the review draws together evidence from type 1 diabetes mellitus (T1DM), type 2 diabetes mellitus (T2DM), impaired glucose tolerance, and dyslipidemia-related stress. We view STZ results as evidence of metabolic injury in insulin-deficient diabetes, not as a full model of human T2DM heterogeneity.

The diabetic biochemical environment provides several routes through which this transport system can fail. Hyperglycemia increases mitochondrial superoxide production and activates major pathways of cellular injury in endothelial cells [9]. Cultured neurons exposed to short-term hyperglycemia show oxidative damage and mitochondrial membrane depolarization. This leads to cytochrome c release, caspase activation, and apoptosis [10]. Diabetes reduces mitochondrial respiratory chain function in DRG, and insulin treatment corrects this deficit in experimental diabetic rats. Impaired signaling through the protein kinase activated by adenosine monophosphate (AMPK) in DRG neurons causes a decline in the mitochondrial respiratory chain function of DRG cells [11]. These mechanisms affect axonal cargo movement, which requires ATP availability, intact cytoskeletal tracks, and calcium homeostasis.

Carbonyl stress and glycation inflict additional layers of damage. Reactive α-dicarbonyls and advanced glycation end products (AGEs) alter neural components–proteins, extracellular matrix, Schwann cells and vascular barriers. Human diabetic sural nerve shows increased glycation of cytoskeletal and myelin protein fractions, with evidence of cytoskeletal protein crosslinking [12]. Experimental diabetes increases glycation of SN cytoskeletal proteins. That is suggesting peripheral nerve proteins are direct targets of chronic glycation [13]. Extracellular matrix proteins modified by AGEs impair sensory neurite outgrowth in vitro, linking glycation of the axonal microenvironment to defective regeneration [14]. The receptor for AGEs (RAGE) can injure primary sensory neurons by oxidative stress, providing a mechanism at the receptor level through which ligand accumulation can be connected with neuronal injury [15].

Inflammation changes the conditions under which sensory neurons maintain long axons. Human DRG from painful DN shows enrichment of inflammatory transcripts, especially transcripts from macrophages, together with reduced neuronal gene expression [16]. Macrophage RAGE activation promotes neuronal insulin resistance and DRG atrophy, and slows retrograde axonal transport [17]. CXCL12–CXCR4 signaling enhances calcium responses and excitability in sensory neurons during DN, linking chemokine activity to altered neuronal function [18]. These observations show that immune and glial activity alters axonal integrity.

Cytoskeletal organization is where these pressures converge. Actin, spectrin, adducin, and related proteins form periodic structures along axons [19]. Disruption of actin structures increases the instability of axonal microtubules, showing that actin organization contributes to microtubule maintenance in axons [20]. DIAPH1, a formin from the diaphanous family, connects Rho family small GTPases with actin remodeling and the organization of the cell [21]. The cytoplasmic tail of RAGE binds DIAPH1, and this interaction is required for ligand-stimulated Rac1 and Cdc42 activation [22]. Structural studies identified a binding region between the RAGE cytoplasmic tail and DIAPH1, providing a molecular route from receptor activation to actin regulatory machinery [23].

**Figure 1:**
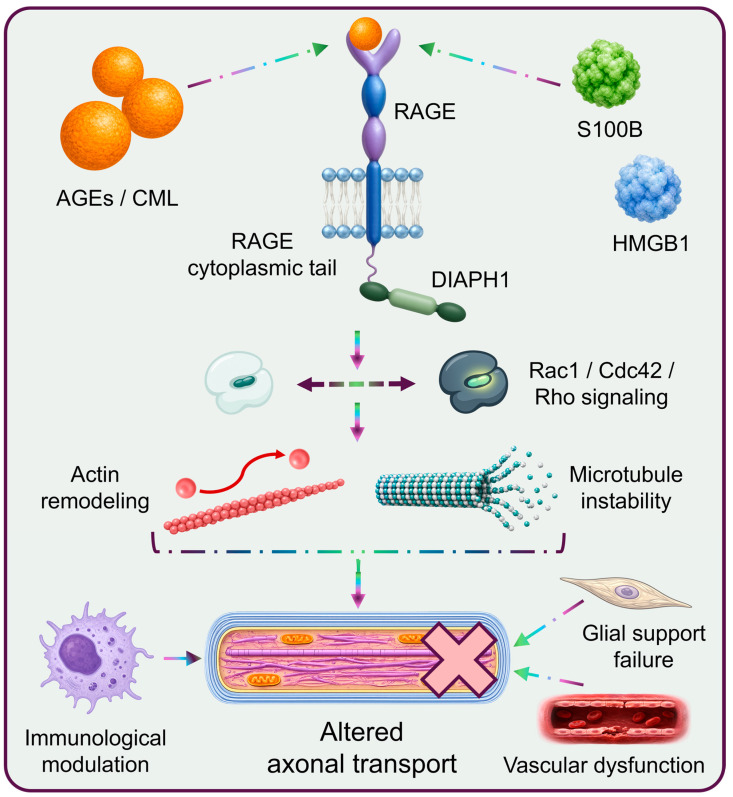
Mechanistic model linking diabetic biochemical stress with axonal transport vulnerability and impaired axonal maintenance. Advanced glycation end products, CML-modified proteins, S100B, and HMGB1 may activate RAGE and promote signaling through the RAGE cytoplasmic tail and DIAPH1. This receptor–effector interaction can engage Rac1, Cdc42, and Rho signaling, thereby influencing actin remodeling and microtubule stability. In parallel, immune activation, vascular dysfunction, and impaired glial support modify the local environment of peripheral axons. Together, these processes may converge on altered axonal transport, impaired cytoskeletal maintenance, and reduced structural integrity of long peripheral axons in diabetic neuropathy.

In this narrative review, the references were chosen to encompass evidence of impaired axonal transport in experimental diabetes, mechanisms of carbonyl stress and AGE–RAGE signaling, RAGE–DIAPH1 coupling, mitochondrial dysfunction, neuroimmune activation, Schwann cell injury, and clinical outcomes of DN. The focus of the review was on axonal maintenance and related processes. This review reframes impaired axonal maintenance as a component of DN, in which carbonyl stress, AGE–RAGE signaling, RAGE–DIAPH1 coupling, mitochondrial dysfunction, neuroimmune activation, Schwann cell injury, and barrier damage converge on cytoskeletal cargo delivery, mitochondrial positioning, and retrograde communication. The conceptual relationship between diabetic biochemical stress, RAGE–DIAPH1 signaling, cytoskeletal remodeling, glial and vascular dysfunction, and impaired axonal transport is summarized in Figure 1.

## 2. Carbonyl Stress and Axonal Dysfunction

Hyperglycemia increases the concentrations of reactive α-dicarbonyls, including methylglyoxal (MGO), glyoxal (GO), and 3-deoxyglucosone (3-DG). This group glycates proteins faster than glucose. Plasma concentrations of α-dicarbonyls are higher [24], and increase more prominently after oral glucose loading in individuals with impaired glucose metabolism [25]. Physiologically, MGO modifies arginine, lysine, and cysteine. In model reactions with N-α-acetylarginine, it first forms reversible glycosylamine and 4,5-dihydroxy-5-methylimidazolidine derivatives. Then it is followed by slower irreversible conversion to an imidazolone adduct. With N-α-acetylcysteine, it rapidly forms a reversible hemithioacetal adduct [26]. Glyoxalase-1 overexpression in endothelial cells reduces intracellular AGE formation during hyperglycemia. It is a demonstration of how detoxification capacity shapes the intracellular load of glycation products [27]. This chemistry relates to neuropathy because impaired dicarbonyl detoxification has been associated with sensory phenotypes. Patients with painful DN show reduced glyoxalase-1 activity compared with diabetic patients without pain symptoms and healthy participants [28]. Glyoxalase-1 expression varies across DRG neuron populations. It is elevated in small sensory neurons, which are important for nociceptive phenotypes [29]. Elevated glyoxalase-1 expression protects diabetic mice from intraepidermal nerve fiber loss and preserves mitochondrial oxidative phosphorylation proteins in DRG [30]. Together, these findings suggest that enhanced dicarbonyl clearance helps in sensory neuron survival, preserves mitochondrial OXPHOS proteins in DRG, and helps maintain distal nerve fibers in diabetes.

AGEs accumulate in patients with clinically measurable neuropathy. In the skin, AGEs deposition precedes and correlates with the clinical manifestations of DN [31]. A clinical study of 111 long-term T1DM patients shows an association between intrinsic skin fluorescence and distal symmetric polyneuropathy and autonomic neuropathy [32]. Another long-term follow-up clinical study of 27 individuals with T1DM confirmed AGEs, including N-ε-carboxymethyllysine (CML) and MGO-derived hydroimidazolone (MG-H1), as correlating with small- and large-fiber dysfunction [33]. In T2DM, skin AGEs discriminate distal sensorimotor polyneuropathy and cardiovascular autonomic neuropathy, including sympathetic and parasympathetic impairment [34]. These clinical associations do not prove axonal transport failure. They, however, show that glycation occurs alongside distal axon failure.

Peripheral nerve tissue contains local evidence of inflammatory signaling connected with glycation. Sural nerve biopsies from neuropathy related to impaired glucose tolerance and DN show CML, RAGE, and NF-κB in the perineurium and in epineurial and endoneurial vessels [35]. Human diabetic peripheral neuropathies (DPN) show increased RAGE, CML, and HMGB1 in peripheral nerve tissue, with mDia1 detectable in both control and neuropathic nerves [36]. In DN, RAGE ligands, activated NF-κB p65, and interleukin-6 (IL-6) colocalize in sural nerve microvessels, and AGE exposure activates NF-κB in SN and DRG through RAGE [37]. These observations suggest that glycation products shape the environment where axons undergo structural remodeling.

Carbonyl stress can also alter axonal physiology through sensory neuron excitability. MGO may increase nociceptive signaling in diabetic neuropathic pain through activation of peripheral TRPA1 and Nav1.8 channels [38]. It activates nociceptors via TRPA1, providing a direct mechanism for pain signaling evoked by carbonyl stress [39]. Human experimental data further indicate that MGO can evoke pain and hyperalgesia through C-fiber activation, with TRPA1 contributing to this response [40]. At low micromolar concentrations, MGO also activates the integrated stress response in IB4-positive DRG nociceptors. ISR inhibition attenuates MGO-evoked and diabetic neuropathic pain in rodent models [41]. Both mechanisms connect carbonyl stress with nociceptor sensitization in diabetic pain models. MGO suppresses TRPM8-mediated cold and menthol responses. That suggests that carbonyl stress can reshape sensory and pain pathways [42]. These mechanisms differ from axonal degeneration, but persistent excitability changes can increase calcium load, energy demand, and mitochondrial stress in vulnerable neurons. MGO also affects mitochondrial and calcium homeostasis, as they relate to transport biology. It alters intracellular calcium signaling, neuronal viability, excitability, and neurite outgrowth in cultured sensory neurons in a concentration-dependent manner [43]. In PC12 cells, MGO increases reactive oxygen species (ROS) and intracellular Ca^2+^. It causes mitochondrial permeability transition, loss of mitochondrial membrane potential, and ultimately apoptosis [44]. In human primary neuron-like cells derived from mesenchymal stem cells, MGO increases ROS and alters glyoxalase expression. It leads to the induction of apoptotic changes and reduces neuronal markers such as MAP2 and neuron-specific enolase [45]. These findings do not directly demonstrate axonal transport failure. However, because intracellular transportation requires ATP and preserved mitochondrial polarization, these effects may reduce transport competence.

Carbonyl and AGE stress directly affect Schwann cells. Methylglyoxal produces oxidative stress and decreases glutathione while activating p38 MAPK and inducing apoptosis [46]. Metformin suppresses MGO-induced apoptosis in mouse Schwann cells and reduces the accumulation of AGEs and ROS [47]. Glycolaldehyde decreases Schwann cell viability at near-physiological concentrations, the antioxidant glutathione; N-acetyl-L-cysteine (NAC), a glutathione precursor; and multidrug resistance-associated protein-1 (MRP1) [48]. Another study links carbonyl stress to endoplasmic reticulum stress and apoptosis. This conclusion is based on the observation that glycolaldehyde also activates PERK, IRE1α, eIF2α, CHOP, caspase-3, and caspase-8 in Schwann cells [49]. AGEs reduce Schwann cell viability, increase apoptosis and ROS, and lower total glutathione. These effects are attenuated by AGER silencing or pharmacological RAGE inhibition [50]. Schwann cells produce myelin and provide trophic and metabolic support. They also regulate the periaxonal environment, and thus their injury can weaken transport and survival even when the initiating lesion is outside the axon.

The blood–nerve barrier is another site where glycation affects axonal health. AGEs decrease claudin-5 expression in microvascular endothelial cells and promote pericyte secretion of VEGF, TGF-β, fibronectin, collagen IV, and TIMP-1. These changes promote disruption of the blood–nerve barrier and hypertrophy of the basement membrane in endoneurial microvessels [51]. According to Toth, MGO compromises the integrity of the human brain endothelial barrier, and edaravone protects against this barrier damage [52]. When the endothelial barrier fails, it disrupts oxygen supply and allows inflammatory cells to enter. In the process, biochemical environment around long peripheral axons changes.

Protein quality control and myelin stability are also affected. SN tissue from db/db mice showed increased protein carbonylation and PMP22 aggregation, with demyelination and slower conduction [53]. In recently diagnosed T2DM, nerve dysfunction related to MGO interacts with magnesium availability, and neuronal models show that magnesium modulates MGO-induced neurite degeneration [54]. The central point is not that carbonyl stress explains every feature of neuropathy. It is that carbonyl stress can disrupt neuronal function from the molecular to the tissue level.

## 3. Signaling Through the RAGE and DIAPH1

RAGE is a transmembrane receptor of the immunoglobulin superfamily, originally cloned as a cellular binding site for AGEs [55]. RAGE, a receptor for advanced glycation end products, has immunoglobulin-like V, C1 and C2 domains, with the V–C1 unit mediating ligand recognition [56]. This architecture enables RAGE to identify different ligands, including glycated proteins, HMGB1, and S100 family proteins [56,57,58]. In DN, this ligand system matters because it can translate injury signals into redox, and transcriptional responses in neurons and cells related to the nervous system.

The diabetic nerve contains several RAGE ligands and signals connected with RAGE activity. Human DPN exhibit elevated levels of RAGE, CML, and HMGB1 in peripheral nerve tissue [36]. Sural nerve biopsies from DN show RAGE ligands, activated NF-κB p65, and IL-6 in peripheral nerve microvessels [37]. Peripheral nerve from neuropathy related to impaired glucose tolerance and DN shows CML, RAGE, and NF-κB in the perineurium and in epineurial and endoneurial vessels [35]. Endoneurial vessels, the perineurium, Schwann cells, macrophages, and axons together form the tissue space in which long axons maintain cargo delivery and distal structural integrity.

RAGE signaling influences axons directly and indirectly. DRG neurons express functional RAGE, and RAGE activation in these neurons induces oxidative stress. It leads to caspase-3 activation, DNA damage, and, in the end, apoptosis [15]. Schwann cells respond to AGEs with reduced viability. ROS accumulate, leading to glutathione depletion, and eventually, increased apoptosis. These effects are attenuated by AGER silencing or pharmacological RAGE inhibition [50]. Macrophage RAGE activation contributes to experimental diabetic polyneuropathy by promoting proinflammatory macrophage profile and impairing neuronal insulin sensitivity. It also reduces DRG neuronal size and slows retrograde axonal transport in the SN [17]. These data support a multicellular model in which RAGE activation damages sensory neurons directly, weakens Schwann cell support, and modifies macrophage behavior in ways that impair axon–soma communication.

The cytoplasmic tail of RAGE is short and lacks an intrinsic catalytic domain. Thus, the intracellular adaptor recruitment is essential for signal propagation [22]. Deletion of the tail blocks neurite outgrowth in neuroblastoma cells, suggesting that it is required for RAGE-driven signaling [59]. The RAGE cytoplasmic domain binds DIAPH1, also known as mDia1, through an interaction involving the formin homology-1 region of DIAPH1 [22]. Reduction in DIAPH1 expression inhibits Rac1 and Cdc42 activation and cell migration following RAGE ligand stimulation. DIAPH1 is placed, therefore, upstream of Rho family GTPase signaling after RAGE engagement [22]. Structural work identified an unusual α-turn in the RAGE cytoplasmic tail that mediates interaction with mDia1 and is required for signaling that depends on RAGE [23]. This makes the RAGE–DIAPH1 interaction a defined receptor–effector mechanism, not a vague downstream consequence of ligand exposure.

Moreover, DIAPH1 can influence microtubule organization. p140mDia, the mammalian homolog of Drosophila diaphanous, acts downstream of Rho small GTPase and binds profilin [21]. Nucleation of actin filaments by formins occurs simultaneously with elongation at the barbed end. Profilin enhances filament elongation by adding profilin–actin complexes [60]. mDia1 can generate actin filaments that resist severing by cofilin, showing that DIAPH1 activity can affect both actin assembly and turnover [61]. In axons, actin turnover contributes to local cargo handling, organelle positioning, growth cone behavior, and the stability of actin structures that support microtubules.

DIAPH1 may also influence microtubule organization. mDia1 coordinates microtubules and F-actin through distinct formin homology regions in cell models [62]. INF2, mDia1, and mDia2 have different effects on microtubules and actin [63]. In cultured hippocampal neurons, pharmacological inhibition of formins modifies actin and microtubule characteristics at the axon initial segment, and mDia1 contributes to the maintenance of axon initial segment composition and structure [64]. Axonal actin, spectrin, adducin, and related proteins form periodic cytoskeletal structures. Disruption of these structures destabilizes axonal microtubules [19,20]. These studies are not specific to diabetes, but they justify the mechanistic inference that RAGE–DIAPH1 signaling may impair transport competence by changing actin dynamics and the interaction between actin and microtubules.

Therapeutic targeting of the RAGE–DIAPH1 interaction strengthens its biological importance. It does not, however, prove that this pathway is the dominant cause of diabetic axonal transport failure. Small molecules that bind the RAGE tail block DIAPH1 interaction and inhibit downstream signaling [65]. RAGE229 lowered FRET between the proteins and improved wound-healing and kidney outcomes in diabetic mice [66]. RAGE406R inhibits DIAPH1 activation via RAGE. The suppression of human macrophage inflammation improved inflammatory and wound-healing phenotypes in experimental models [67]. These studies show that this receptor and its partner can be blocked by small molecules.

Recent DN models make the interpretation more specific. Global Diaph1 deletion does not entirely stop the progression of DPN or fully rescue nerve conduction defects, arguing against DIAPH1 as a single sufficient explanation for diabetic nerve injury [68]. In diabetic mice lacking both RAGE and DIAPH1, interruption of RAGE–DIAPH1 signaling altered actin regulatory balance involving cofilin and profilin, improved axonal structure and motor nerve conduction velocity in the SN [69]. RAGE–DIAPH1 signaling is a plausible mechanism for cytoskeletal regulation, but it has not been shown to drive diabetic axonal transport failure. Current evidence does not demonstrate that selective manipulation of RAGE–DIAPH1 signaling restores axonal transport dynamics in diabetic peripheral axons.

RAGE–DIAPH1 signaling should thus be considered a connector between diabetic ligand accumulation and cytoskeletal injury. AGEs, CML-modified proteins, HMGB1 and S100 proteins activate RAGE in peripheral nerves [15,17,35,36,37,50]. The RAGE cytoplasmic tail can recruit DIAPH1 and activate small GTPase signaling, while DIAPH1 can regulate actin assembly, actin turnover, and coordination between actin and microtubules [21,22,23,60,61,62]. RAGE–DIAPH1 signaling should be considered a mechanistically attractive route into cytoskeletal regulation, not yet an established pathway of diabetic axonal transport failure. The main mechanistic links connecting diabetic biochemical stress, AGE–RAGE signaling, RAGE–DIAPH1 coupling, mitochondrial dysfunction, Schwann cell injury, neuroimmune activation, and cytoskeletal instability with axonal maintenance are summarized in Table 1.

## 4. Oxidative Stress and Mitochondrial Dysfunction

Axonal transport is energetically demanding. Molecular motors move structural and functional structures and damaged material across long distances. Kinesin acts as a force-producing protein that moves along microtubules. Cytoplasmic dynein was established as a major motor for retrograde axonal transport [71,72]. Mitochondria move in axons along microtubules and F-actin. That places mitochondrial positioning under direct control of cytoskeletal organization [73]. These systems require ATP, controlled calcium signaling, intact microtubule tracks, and mitochondrial support at specific locations. Diabetes threatens axonal maintenance by damaging both the energy supply and the structural tracks on which transport depends.

Hyperglycemia increases ROS production through several mechanisms. In vascular endothelial cells, high glucose increases mitochondrial superoxide production. This contributes to protein kinase C activation, AGE formation, sorbitol accumulation, and NF-κB activation [9]. In neurons, short-term hyperglycemia inflicts oxidative damage, which triggers mitochondrial membrane depolarization and cytochrome c release. The following caspase activation leads to DNA fragmentation and apoptosis [10]. These changes pertain to transport biology because mitochondrial depolarization limits ATP production. Meanwhile, caspase activation marks transition from reversible metabolic stress toward structural damage.

DRG neurons show direct mitochondrial respiratory impairment in diabetes. STZ-induced diabetes reduces mitochondrial respiration in DRG, and insulin treatment corrects this defect [70]. Diabetes also suppresses mitochondrial respiratory chain protein expression in DRG, linked to impaired AMPK and PGC-1α signaling. Changing AMPK activity alters neurite outgrowth directed by neurotrophins in adult sensory neurons, thereby links cellular energy sensing to the neuron’s ability to grow [11]. Distal axons need constant ATP to power transport and maintenance. Impaired AMPK-PGC-1α signaling may reduce transport competence before axonal degeneration becomes morphologically observable.

PGC-1α helps in maintaining mitochondrial integrity, while loss of PGC-1α causes their breakdown, resulting in lower respiration and weaker antioxidant responses. In the opposite situation, PGC-1α overexpression protects neurons from oxidative damage caused by high-glucose [74], demonstrating a relationship between impaired mitochondrial biogenesis and insufficient antioxidant transcriptional responses, which make sensory neurons less able to maintain the mitochondrial pool required for long axons. The link to transport is partly inferential: mitochondrial number, respiratory capacity, and oxidative protein damage determine where to place and use the organelles in distal axonal regions.

Mitochondrial dynamics are altered during diabetic neuronal injury. DRG neurons from diabetic mice show increased mitochondrial biogenesis with small, fragmented mitochondria, and short-term hyperglycemia increases dynamin-related protein 1 (DRP1) levels in cultured neurons [75]. Drp1 knockdown decreases susceptibility to hyperglycemic damage in this model, showing that excessive mitochondrial fission contributes to neuronal vulnerability [75]. Mitochondrial calcium uniporter deletion prevents painful DN in mice by restoring mitochondrial morphology and dynamics [76]. These findings do not prove a direct transport defect. However, they indicate that diabetes alters mitochondrial shape, calcium handling, and damage responses.

Nitrosative stress exacerbates damage to energy-producing components of diabetic nerves. Peroxynitrite decomposition reduces nitrotyrosine and poly(ADP-ribose) accumulation in SN, spinal cord, and DRG neurons of STZ diabetic mice and improves large motor and sensory, and small sensory fiber function [77]. PARP inhibition in STZ-diabetic rats counteracts motor and sensory nerve conduction slowing, large myelinated fiber atrophy, and increased nitrotyrosine and tumor necrosis factor-α (TNF-α) in the SN and spinal cord [78]. PARP activation depletes NAD+ and disrupts energy metabolism, thereby contributing to axonal dysfunction.

AGE–RAGE signaling contributes to oxidative stress inside sensory neurons. Primary DRG neurons express functional RAGE, and RAGE activation triggers phosphatidylinositol 3-kinase activity, which leads to ROS production, then caspase-3 activation and DNA degradation [15]. Antioxidants block these effects, indicating that RAGE-induced oxidative stress damages sensory neurons [15]. In DN, AGE exposure activates NF-κB in SN and DRG through RAGE [37]. Redox and inflammatory signaling through RAGE may impair axonal transport indirectly by damaging mitochondria and directly by altering the cytoskeletal environment in which motor and cargo proteins operate.

When Schwann cell mitochondria are damaged, they cannot support axonal health even if the axon itself was not damaged. Intact Schwann cell mitochondrial metabolism is required for long-term axonal survival and peripheral nerve function [79], while hyperglycemia alters their mitochondrial respiration and proteome [80]. Disrupted Schwann cell lipid metabolism produces peripheral neuropathy with axonal degeneration, associated with mitochondrial dysfunction and altered glial metabolic support [81]. In diabetes, Schwann cell mitochondrial injury may reduce the external metabolic support required for sustained axonal cargo movement.

Oxidative damage to proteins may also weaken the cytoskeletal and motor systems that underlie axonal transport. SN from db/db mice shows increased protein carbonylation and aggregation of peripheral myelin protein 22 (PMP22), accompanied by demyelination and reduced nerve conduction velocity [53]. Protein carbonylation and nitrotyrosine formation indicate oxidative and nitrosative modification of proteins in diabetic nerve compartments [53,77]. If cytoskeletal proteins, motor proteins, adaptor complexes, or mitochondrial proteins undergo such modifications, transport competence may decline before axonal transection occurs. The connection requires investigation to move from mechanistic inference to confirmed evidence.

Mitochondrial dysfunction and transport failure can amplify each other. Impaired mitochondrial movement reduces ATP availability and calcium buffering at distal axonal sites [73]. Lower ATP also halts motor protein-mediated cargo movement, ion pump activity, cytoskeletal renewal, and local repair. Oxidative stress damages mitochondrial proteins and may destabilize microtubules and actin structures that guide cargo movement. In this model, mitochondrial dysfunction may participate in the cellular processes that link metabolic stress to distal axonal degeneration, impaired regeneration, and sensory dysfunction.

## 5. Neuroimmune Activation Changing the Axonal Environment

Neuroimmune activation in DN is best understood as a change in the axonal environment. Human L4 and L5 DRG from patients with painful DN show increased expression of transcripts that are involved in inflammation, particularly those from macrophages, with simultaneous downregulation of multiple neuronal genes [16]. This pattern has direct implications for axonal biology. DRG contain the sensory neuron somata that synthesize, sort, and export cytoskeletal proteins, organelles, ion channel components, trophic receptors, and signaling cargoes into long peripheral axons. Inflammation in this compartment may thus affect the transport at its source.

Systemic inflammatory biomarkers support a link between DN and immune activation, although they do not localize the process to axons or ganglia. In the KORA F4 cohort, subclinical inflammation was related to prevalent polyneuropathy in an older population [82]. In a prospective cohort of patients with T2DM, higher plasma levels of proinflammatory factors, especially TNF-α and intercellular adhesion molecule-1, predicted incident diabetic peripheral neuropathy over five years [83]. Although serum TNF-α is elevated in T2DM and correlates with nerve conduction abnormalities [84], this provides clinical context and not direct evidence that cytokines impair axonal transport.

Macrophage activation provides a stronger experimental link between inflammation and transport failure. In diabetic mice, infiltrating proinflammatory macrophages impair insulin sensitivity, reduce DRG neuronal size, and slow retrograde axonal transport in the SN. RAGE-null mice maintain insulin sensitivity, normal ganglion cell size, and intact retrograde transport. A study showed that RAGE-null bone marrow partially protected diabetic mice from peripheral nerve deficits, indicating that macrophage RAGE contributes to the inflammation that disrupts neural maintenance [17]. Retrograde axonal transport also carries signals from distal axons to the neuronal soma. This process allows neurons to adjust transcription and repair responses. In diabetic mice, proinflammatory macrophage infiltration accompanies impaired insulin-AKT-GSK3β signaling in peripheral nerves, while bone marrow-specific RAGE deletion preserves this pathway and improves retrograde transport [17]. This suggests a sequence in which macrophage and receptor activation, as well as local insulin resistance impair retrograde signaling that supports axon survival.

Satellite glial cells directly envelop DRG neuron somata. STZ-treated rodents show elevated GFAP and more neurons encircled by activated glia. Activated glia alter the cell-body environment and change how cytoskeletal proteins, organelles, and receptor complexes are prepared for axonal transport [85].

A more specific pathway involving lipocalin-2 has been characterized. Diabetes increases lipocalin-2 in satellite glia, and lipocalin-2 increases pyruvate dehydrogenase kinase-2 expression through PPARβ/δ. This inhibits pyruvate dehydrogenase activity and increases lactic acid production in DRG satellite glia and neurons. Genetic and cellular experiments support the lipocalin-2–PPARβ/δ–PDK2 pathway, which increases lactic acid [86]. This pathway links glial inflammation with altered energy metabolism in the ganglion compartment that supplies long axons.

Chemokines connect immune activation with sensory neuron excitability and ganglion inflammation [18]. CCL2–CCR2 signaling may also contribute to neuron-immune communication. In STZ-diabetic rats with diabetic gastropathy, CCR2 is upregulated in DRG neurons and contributes to gastric hyperalgesia [87]. For somatic distal polyneuropathy, this evidence remains less direct than the CXCL12–CXCR4 data, so CCL2–CCR2 should be considered a plausible pathway.

Other pain and inflammatory amplification pathways demand greater caution. In T2DM rodents with painful neuropathy, HMGB1, TLR4, CXCR4, and NLRP3 are increased in the spinal cord and DRG. Glycyrrhizin treatment reduces these inflammatory markers while improving pain thresholds [88]. In STZ-diabetic mice, the NLRP3 pathway activates in DRG, and pharmacological inhibition of NLRP3 with MCC950 reduces mechanical allodynia [89]. STZ-diabetic rats with mechanical hyperalgesia show spinal MAPK activation dependent on N-methyl-D-aspartate receptors in neurons and microglia [90]. These findings clarify inflammatory amplification and pain signaling; however, they do not directly address axonal transport. They are therefore used here as evidence of an inflammatory environment that may influence axonal transport, not as direct evidence of impaired cargo movement. Future work should test links to motor proteins or cytoskeletal cargo delivery.

Central microglial activation should be considered a secondary concern. Spinal microglia are activated in diabetic pain models and can release cytokines and neuroactive mediators that increase dorsal horn excitability [91]. In experimental diabetic encephalopathy, modulation of IL-17A signaling reduced neuroinflammation and cognitive impairment, supporting a link between diabetes, central inflammation, and neuronal dysfunction [92]. Macrophage RAGE activation impairs retrograde transport and neuronal insulin signaling. Satellite glial activation alters the ganglion environment in which neuronal somata maintain axonal export. Chemokine signaling increases sensory neuron calcium responses and energy demand. Lastly, HMGB1, Toll-like receptors, MAPKs, and NLRP3 mainly support a model of pain and inflammatory amplification indirectly relevant to transport.

## 6. Axonal Cytoskeletal Remodeling and Transport Failure: Direct Evidence and Mechanistic Inference

Throughout this review, we try to emphasize that not every diabetic mechanism that damages nerves, impairs cargo movement as well. The interpretation of neuroinflammation should be precise and multilayered. We interpret evidence on three levels: direct transport measurements; mechanisms that may affect transport machinery; and clinical or structural readouts of distal axonal maintenance. Some studies inspect transport. Others prove the biological underpinnings of transport failure by demonstrating mitochondrial and cytoskeletal damage, glial support, or retrograde signaling impairment.

Long peripheral axons require uninterrupted delivery of axonal cargo from the soma to distal axon regions. Slow axonal transport carries cytoskeletal and soluble proteins. Meanwhile, fast transport moves membranous organelles and signaling complexes along polarized microtubules. Neurofilament movement is not a continuous slow flow. Live imaging shows bursts of movement, explaining how a slowly advancing population can emerge from brief events driven by motor proteins [93]. Subtle disturbances in motor function, cytoskeletal tracks, and cargo assembly may reduce distal renewal before axonal transection is visible.

The strongest direct evidence comes from experimental diabetes models, which show a reduced slow transport of cytoskeletal elements. An early experimental study shows that mutant diabetic mice exhibit altered axonal transport of actin, tubulin, and neurofilament proteins [4]. STZ-diabetic rats show impaired slow transport of cytoskeletal proteins in the SN after injection of a radiolabeled precursor into the lumbar ganglia and the spinal cord [5]. Similar slow transport abnormalities and axonal size changes were observed across different DN models, supporting a relationship between transport impairment and axonal atrophy [3]. These studies view DN as a problem of axonal maintenance, and not just as a conduction or microvascular issue.

Progressive diabetes results in altered cytoskeletal protein synthesis and export. In STZ-diabetic rats, progressive disease decreases neurofilament and tubulin mRNA expression in DRG and lowers incorporation of these proteins into distal sensory axons [7]. Sensory neuron bodies in DRG are the source of the cytoskeletal proteins that sustain long peripheral axons. Reduced synthesis and reduced export thus provide a mechanistic explanation for distal decline in fiber diameter. Chronically STZ-diabetic rats show reduced myelinated fiber size, and the reduction in their fiber diameter correlates with the loss of axonal neurofilaments [6]. Since neurofilaments are major constituents in myelinated fibers, their depletion links impaired cytoskeletal supply with reduced fiber diameter and conduction dysfunction.

Diabetes also impairs transport of nonstructural metabolic cargoes. STZ-diabetic rats show impaired accumulation of phosphofructokinase activity on both sides of SN constrictions, and this defect is not corrected by sorbinil despite suppression of sorbitol and fructose accumulation in SN [94]. Diabetes interferes with axonal transport of glycolytic enzymes via mechanisms beyond the aldose reductase pathway. Retrograde transport is another mechanism affected by diabetes. STZ-diabetic rats show impaired retrograde axonal transport after SN crush, as reflected by altered accumulation of labeled protein and glycoprotein markers after precursor injection into lumbar spinal ganglia [95]. More recent work links macrophage RAGE activation with reduced neuronal insulin sensitivity, reduced DRG neuronal size, and slowed retrograde axonal transport in SN [17].

Fast transport defects are cargo-specific. In STZ-diabetic rats, phosphorylated JNK and p38 are elevated in the SN. However, total JNK and p38 transport, as well as anterograde transport of phosphorylated JNK and p38, are not significantly altered [96]. This does not show a global reduction in fast transport. It shows that diabetes changes the retrograde movement of activated stress kinases, which may influence transcriptional stress responses, cytoskeletal regulation, and survival programs. There is less information about motor protein changes in peripheral DN. Altered KIF1A and KIF5B expression has been reported in hippocampal tissue and high-glucose neuronal cultures, but these findings are best considered auxiliary evidence that diabetes can alter transport systems outside peripheral nerves [97].

Mitochondrial trafficking connects axonal transport to energy disturbances. Mitochondria move in vertebrate axons along microtubules and F-actin, making their position dependent on microtubule tracks and actin structures [73]. Dyslipidemia impairs mitochondrial trafficking and function in sensory neurons, which matters for T2DM, since dyslipidemia is a significant metabolic factor for neuropathy risk [98]. High dietary fat intake impairs axonal mitochondrial function in vivo, providing additional evidence that metabolic stress can injure the axonal energy system at the level of the axon itself [99]. These studies extend the model beyond hyperglycemia, but they do not replace direct transport studies in diabetic peripheral nerve.

Regeneration failure shows the functional consequence of defective cytoskeletal remodeling. After nerve crush, diabetic rats show altered tubulin and neurofilament expression, reduced axonal elongation and fiber-caliber growth of regenerating fibers [8]. Regeneration depends on tubulin for microtubule extension, actin remodeling at growth cones, incorporation of neurofilaments, and mitochondrial redistribution for local ATP. Diabetes can impair regeneration through the same transport and cytoskeletal systems that sustain uninjured distal axons.

The axonal cytoskeleton should be considered as an integrated system of actin, microtubules, neurofilaments, and membrane–skeleton elements. Super-resolution microscopy shows that actin, spectrin, adducin, and related proteins form a periodic structure along axons, with actin rings spaced at ~180–190 nm [19]. Periodic actin structures maintain axonal microtubules, and their disruption destabilizes the microtubule network [20]. These observations are not diabetes-limited, but they are crucial for understanding diabetic cytoskeletal damage. Impaired actin regulation could secondarily disturb microtubule organization and compromise both slow and fast transport.

DIAPH1 connects RAGE signaling to the actin cytoskeleton. p140mDia functions downstream of Rho small GTPase and interacts with profilin [21]. Formins nucleate and elongate actin filaments at their barbed ends, while profilin speeds this reaction by delivering profilin–actin complexes [60]. mDia1 can make filaments that resist cofilin severing, showing it affects both assembly and turnover [61]. These mechanisms provide a plausible molecular route through which RAGE–DIAPH1 signaling could modify actin dynamics in diabetic nerve, but they do not prove that DIAPH1 directly controls axonal transport in human DN.

Coordination between actin and microtubules provides a second route through which DIAPH1 may affect axonal transport. mDia1 coordinates microtubules and F-actin through distinct formin homology regions in cell models [62]. The RAGE cytoplasmic domain binds DIAPH1, and this interaction is required for RAGE ligand-stimulated Rac1 and Cdc42 activation [22]. Possibly, diabetic RAGE ligands activate RAGE, recruit DIAPH1, stimulate Rho family GTPase signaling, and alter coordination between actin and microtubules. It is still largely unknown, how this affects axonal transport, until studies directly show transport and mitochondrial measurements after selective manipulation of RAGE–DIAPH1 signaling.

Recent mouse studies refine this point. Global Diaph1 deletion does not stop diabetic peripheral neuropathy progression, arguing against DIAPH1 as a single essential cause of diabetic nerve injury [68]. In diabetic mice RAGE and Diaph1 knockouts, interruption of RAGE–Diaph1 signaling improves axonal structure and motor nerve conduction velocity, with changes in actin regulatory balance involving cofilin and profilin [69]. These results support the mechanistic role of the RAGE–Diaph1 pathway in regulating axonal actin under chronic hyperglycemia. They also justify placing this pathway within a larger model that includes mitochondrial dysfunction, carbonyl stress, macrophage activation, Schwann cell dysfunction, and barrier injury.

A model organized around axonal transport thus separates demonstrated mechanisms from inferred mechanisms. Diabetes directly decreases synthesis and export of neurofilaments and tubulin; reduces neurofilament content and axonal diameter; impairs transport of phosphofructokinase; alters retrograde stress signaling and weakens regeneration. General axonal biology shows that neurofilament movement, mitochondrial positioning, actin–spectrin organization, and microtubule integrity are interdependent systems required for axonal maintenance. RAGE–DIAPH1 signaling provides a pathway from ligand detection to actin regulation, but a direct causal link to axonal transport failure in human diabetes has not been established. The experimental evidence linking diabetes with impaired axonal transport, cytoskeletal cargo delivery, retrograde signaling, and regenerative failure is summarized in Table 2.

## 7. Peripheral and Autonomic Axonopathy in Diabetes

Distal symmetric polyneuropathy is the primary clinical manifestation of diabetic axonopathy. DN usually follows a distal pattern that depends on fiber length, in which the longest peripheral axons are especially vulnerable to chronic metabolic stress [1,100]. This does not mean that injury to large myelinated fibers necessarily precedes injury to small fibers in every patient. Small unmyelinated C fibers and thinly myelinated Aδ fibers may show structural or functional abnormalities that are not detected by conventional nerve conduction studies, because these studies mainly assess large myelinated fibers [101]. Longitudinal studies further indicate that small- and large-fiber measures may progress differently, depending on diabetes type, phenotype, and the method used for detection [102,103]. Large myelinated fibers also become involved during disease progression and contribute to the phenotype through reduced amplitudes, conduction slowing, nodal or paranodal dysfunction, and myelin abnormalities [104,105]. Thus, the early sequence of neuronal involvement should be interpreted as distal axonal vulnerability that depends on fiber length, with phenotype- and method-dependent involvement of small and large fibers, rather than as a strict sequence in which one fiber class is always affected first.

Nerve conduction studies evaluate large-fiber function using amplitude measures together with latency and conduction velocity. Electrodiagnostic patterns in diabetic sensorimotor polyneuropathy include conduction slowing and reduced amplitudes [104]. Conduction slowing indicates that myelin, nodal, paranodal, or axo-glial dysfunction can contribute to the phenotype, while reduced amplitudes more directly reflect axonal loss [104]. DN is not purely an axonal disorder. It affects the axon–glia–vascular unit, in which axonal transport failure, myelin damage, Schwann cell dysfunction and vascular changes intersect during disease progression [3,5,7,8,51,79,80,81,105,106].

Small fiber degeneration may be detected before conventional large-fiber measures become abnormal. In patients with diabetes, reduced intraepidermal nerve fiber density and abnormal thermal thresholds can be present despite normal results in conventional nerve conduction studies, supporting early small-fiber damage that may remain undetected by standard nerve conduction tests [101]. In T2DM, serial distal-leg biopsies show progressive loss of intraepidermal fibers at a faster rate than in healthy controls [102]. Over five years, the decline in small-fiber measures was more pronounced than the decline in large-fiber measures, and neuropathy progression was greater in T2DM than in T1DM [103]. Intraepidermal nerve fibers represent terminal distal axonal compartments, making their loss a structural readout of failed distal axonal maintenance [101,102,103].

Corneal confocal microscopy provides another structural window into small-fiber degeneration. Corneal nerve length was shown to predict diabetic peripheral neuropathy in a longitudinal multinational cohort [107]. The corneal subbasal nerve plexus contains small sensory axons that can be imaged noninvasively and measured over time. Reduced corneal nerve fiber length can thus be interpreted as a measurable marker of small axon integrity, not only as an ophthalmic feature of diabetes. More recent comparative work indicates that corneal nerve fiber measures and intraepidermal nerve fiber density can both detect small-fiber neurodegeneration in T2DM, although their relationship with clinical neuropathy may differ by cohort and endpoint [108]. Viewed through an axonal-transport model, these measures track distal axonal integrity over time. They do not directly measure axonal transport, but they capture distal axonal integrity, which is the expected tissue-level consequence of impaired axonal maintenance.

Sudomotor dysfunction adds an autonomic small-fiber dimension. Electrochemical skin conductance (ESC) measured by Sudoscan is lower in diabetic patients with neuropathy compared with diabetic patients without neuropathy or healthy controls. Foot ESC shows diagnostic performance for diabetic neuropathy (AUC ≈ 0.88) [109]. Electrochemical skin conductance is a measure of how easily electricity passes through the skin. Casellini found it relates to clinical, sensory, autonomic, and pain measures [109]. Intrinsic skin fluorescence is associated with both autonomic neuropathy and confirmed distal symmetrical polyneuropathy in T1DM [32]. These data support inclusion of autonomic readouts, but they should serve the main neuropathy argument rather than redirect the review toward cardiac disease.

DRG house the sensory neuron cell bodies that make structural and functional molecules, which peripheral axons need. In diabetic BioBreeding/Worcester rats, a reduction to 73% of normal DRG neurons was observed, with selective loss of substance P– and CGRP-positive neurons, slowed sensory nerve conduction, fewer sural myelinated and unmyelinated fibers, and progressive Golgi degeneration [110]. Injured Golgi is especially important because axonal cargo supply depends on neuronal protein processing and sorting before export into the axon.

Human DRG data also support proximal sensory neuron involvement. Human L4–L5 DRG samples from patients with painful DN show increase in inflammation-related macrophage transcripts and decreased neuronal gene expression [16]. Three Tesla magnetic resonance neurography shows that diabetic polyneuropathy is related to pathomorphological changes in human DRG, and DRG signal intensity correlates with neuropathy severity and metabolic measures [111]. Human DRG from donors with diabetic peripheral neuropathy contain abundant Nageotte nodules, which are linked to sensory neuron death and dystrophic nociceptive axons [112]. These findings place DN within a sensory neuron-satellite glia-ganglion compartment, not only within the distal nerve trunk.

Schwann cell and myelin abnormalities modify the axonal phenotype. Human DN shows histopathological heterogeneity, including axonal degeneration, segmental demyelination, remyelination, and axo-glial dysjunction [105]. In diabetic mice, blockade of mixed lineage kinase domain-like protein prevents myelin decompaction. It also attenuates reduction in nerve conduction velocity caused by diabetes, supporting a role for Schwann cell necroptosis and myelin injury in the functional neuropathy phenotype [106]. These observations show that axonal transport depends on the integrity of the Schwann cell, myelin sheath, node, paranode, and periaxonal metabolic environment.

Central nervous system manifestations should be included sparingly. In STZ-diabetic rats, diabetic encephalopathy is accompanied by mitochondrial changes and altered hippocampal expression of axonal transport proteins, including increased KIF5b mRNA [113]. This does not imply equivalence. It rather indicates that diabetes may affect neuronal compartments involved in cognition via mitochondrial and transport mechanisms. Such data can support the general concept of neural injury in different compartments, but the primary focus should remain peripheral sensory and autonomic axons.

The clinical measures that align best with the current model are those that measure axonal integrity, fiber loss, conduction, and small-fiber function. Nerve conduction studies, which assess large-fiber function, can differentiate axonal loss (reduced amplitudes) from conduction slowing [104]. Skin biopsy counts intraepidermal nerve fibers to assess distal small-fiber integrity [101,102]. Corneal confocal microscopy measures small sensory axons in the cornea and predicts incident diabetic peripheral neuropathy [107]. Quantitative sensory testing, sudomotor testing, heart rate variability, and cardiovascular autonomic reflex testing provide functional complements [1,109]. Together, these measures translate axonal maintenance failure into clinically observable phenotypes.

## 8. Therapeutic Strategies and Translational Readouts for Axonal Preservation

**Figure 2:**
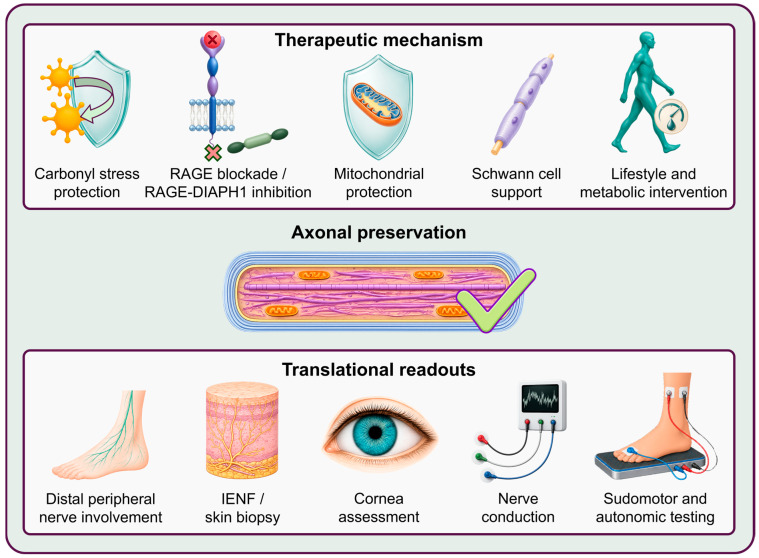
Therapeutic strategies and translational readouts for axonal preservation in diabetic neuropathy. Candidate strategies include reduction of carbonyl stress, blockade of RAGE signaling or inhibition of the RAGE–DIAPH1 interaction, mitochondrial protection, Schwann cell support, and lifestyle or metabolic interventions. These approaches are conceptually organized around preservation of axonal structure, mitochondrial competence, cytoskeletal integrity, and glial support. Translational assessment should combine structural and functional readouts, including distal peripheral nerve involvement, intraepidermal nerve fiber assessment in skin biopsy, corneal nerve assessment, nerve conduction studies, sudomotor testing, and autonomic testing.

A therapy oriented to protect axonal maintenance would be expected to preserve cytoskeletal integrity, mitochondrial health, axon-to-soma signaling, and the glial and vascular support of long axons. Symptomatic analgesia improves quality of life, but it does not necessarily address underlying issues. This therapeutic section includes several interventions with symptomatic, metabolic, or structural effects, but the direct relationship of these interventions to preservation of axonal transport remains incompletely established. The therapeutic logic of axonal preservation and the corresponding translational readouts are outlined in Figure 2.

Improved metabolic control remains necessary, but it is not sufficient as a strategy for transport preservation. The one-year study in patients with impaired glucose tolerance and neuropathy showed that individualized diet and exercise counseling increased distal and proximal intraepidermal nerve fiber density and reduced neuropathic pain [114]. In patients with diabetes without neuropathy, supervised exercise increased cutaneous nerve density over 12 months, while standard counseling was accompanied by stasis or decline in intraepidermal nerve fiber density [115]. The findings indicate that metabolic and activity-based treatments can improve the distal axonal compartment.

Carbonyl stress reduction is an upstream strategy because MGO, glyoxal, 3-deoxyglucosone, and AGEs modify proteins that support axonal structure, mitochondrial function, extracellular matrix permissiveness, and sensory neuron excitability. Research indicates that diabetic mice with increased glyoxalase-1 show protection against intraepidermal nerve fiber loss while maintaining mitochondrial oxidative phosphorylation proteins in DRG [30]. MGO may contribute to diabetic neuropathic pain through activation of peripheral TRPA1 and Nav1.8 channels in sensory neurons [38]. No direct human data demonstrate restoration of slow axonal transport or mitochondrial trafficking; future work should combine protein–carbonyl assays with skin biopsy quantification of intraepidermal nerve fiber density and nerve conduction or trafficking assays.

MGO scavengers and inhibitors of AGE formation should be interpreted with similar caution. Aminoguanidine improves motor nerve conduction velocity and limits structural abnormalities in experimental DN. Older preclinical evidence, however, does not establish clinical efficacy for human axonal preservation [116]. Extracellular matrix proteins modified by AGEs impair sensory neurite outgrowth. The therapies that prevent matrix glycation or remove glycated matrix constraints might enhance regeneration [14]. The problem with translational research is that most carbonyl scavenger or AGE inhibitor studies do not assess slow and fast axonal transport, motor protein function, and mitochondrial trafficking.

RAGE blockade is most convincing when linked to neuronal, glial, immune, or vascular mechanisms that influence axonal maintenance. RAGE activation injures primary sensory neurons through oxidative stress and apoptotic signaling [15]. RAGE activation in macrophages contributes to experimental diabetic polyneuropathy, neuronal insulin resistance, DRG atrophy, and slowed retrograde axonal transport [17]. These data make RAGE a plausible target for preserving axon–soma communication and the inflammatory environment of the sensory ganglion. RAGE inhibition isn’t panacea–carbonyl stress; mitochondrial defects, Schwann cell injury, dyslipidemia, barrier dysfunction, and transport defects can persist independently of RAGE.

In cell and animal models, small molecules that disrupt the RAGE–DIAPH1 interaction inhibit RAGE signaling after ligand stimulation [65]. RAGE229 reduces the RAGE–DIAPH1 interaction and attenuates diabetic complications in mice, including impaired wound healing and kidney injury [66]. RAGE406R prevents DIAPH1 activation via RAGE and suppresses human macrophage inflammatory responses [67]. Evidence for neuropathy remains preclinical. Future studies should explore the relationships between cytoskeletal proteins and slow axonal transport.

Antioxidant and mitochondrial interventions illustrate why endpoint choice is important. In the SYDNEY 2 trial, oral α-lipoic acid (ALA) improved neuropathic symptoms and deficits over five weeks in patients with diabetic sensorimotor polyneuropathy. A dose of 600 mg daily yielded the best risk–benefit profile [117]. In the NATHAN 1 trial, four years of ALA did not significantly improve the primary composite endpoint in mild-to-moderate diabetic sensorimotor polyneuropathy. Yet, it produced clinically meaningful improvements in neuropathic impairments and had acceptable safety [118]. Acetyl-L-carnitine improved pain, nerve fiber regeneration, and vibration perception in two randomized placebo-controlled trials of chronic DN [119]. In T1DM, oral omega-3 fatty acid supplementation increased central corneal nerve fiber length over six months in a randomized placebo-controlled trial [120]. These interventions are consistent with axonal-transport models but do not directly target motor proteins or mitochondrial movement. Because oxidative stress, neuroimmune activation, and Schwann cell injury can converge on axonal maintenance, therapies directed at these processes should be assessed beyond pain behavior and inflammatory markers. Sulforaphane modulates Nrf2 and NF-κB signaling and counteracts several manifestations of experimental DN in rats [121]. Another study measures vascularity, nerve density, and nerve conduction in DPN models with nerve injury and reports that Schwann cells overexpressing Nrf2 enhance sciatic nerve recovery [122]. These strategies diminish glial inflammation and neuron sensitization. Most studies report pain behavior and inflammation, and only a few directly measure axonal transport or mitochondrial trafficking. This limitation should be considered when interpreting symptomatic or anti-inflammatory improvement as evidence for axonal preservation. Schwann cell preservation is important because axons depend on glial support for myelin integrity and the intra- and periaxonal environment. Hyperglycemia alters Schwann cell mitochondrial respiration and proteome [80]. It is vital because Schwann cell mitochondrial metabolism supports long-term axonal survival and peripheral nerve function [79]. Blockade of mixed-lineage kinase domain-like protein prevents myelin decompaction and attenuates reduced nerve conduction velocity in experimental DN [106]. Therapies that protect Schwann cell mitochondria, myelin architecture, and glial survival could indirectly preserve axonal transport by maintaining the axon’s external support system. Regenerative therapies should be assessed by their ability to restore axonal structure and function. The 8% capsaicin patch is used as a topical analgesic for painful diabetic peripheral neuropathy. A clinical skin biopsy study reported increased intraepidermal and subepidermal nerve fiber density after treatment. The effects were accompanied by pain reduction and improved warmth perception [123]. This observation links analgesia with structural changes in small fibers. It requires cautious interpretation and confirmation in larger, prespecified studies with longer follow-up. The therapeutic and translational evidence discussed above can be organized according to the level of intervention, measured endpoint, and relevance for axonal preservation. These strategies and readouts are summarized in Table 3.

The most useful translational endpoints are those that match the biological requirements of axonal transport and maintenance. Nerve conduction studies assess large-fiber function. Axonal loss, reflected by reduced amplitudes, can be distinguished from conduction slowing. Skin biopsy enables direct measurement of intraepidermal nerve fiber density. Corneal confocal microscopy quantifies corneal nerve fiber length and related small-fiber measures and can detect early regeneration or disease progression. Quantitative sensory testing, sudomotor testing, and autonomic reflex tests provide functional complements to structural readouts. Mechanistic trials should combine clinical endpoints with biomarkers that assess methylglyoxal exposure and glyoxalase activity, AGE formation and soluble RAGE, and markers of oxidative damage, mitochondrial function, and inflammation.

Axonal transport impairment should be interpreted as one component of DN pathogenesis, not as a replacement for established vascular, glial, metabolic, and immune mechanisms. AGE exposure can disrupt the blood–nerve barrier and remodel endoneurial microvessels [51], while Schwann cell injury can affect myelin integrity, mitochondrial support, and the periaxonal environment required for long-term axonal survival [79,80,81,105,106]. Metabolic stress also contributes to injury of sensory neurons and axonal mitochondria through mitochondrial respiratory impairment, altered AMPK signaling, oxidative damage, mitochondrial fission, and injury associated with dyslipidemia [9,11,70,75,98,99]. In parallel, immune activation in DRG and peripheral nerves may alter neuronal soma, macrophage behavior, insulin signaling, and the local inflammatory environment, thereby affecting axonal cargo delivery and retrograde communication [16,17,82,83]. These mechanisms may affect axonal transport by limiting local ATP availability, cytoskeletal stability, cargo handling, and glial or vascular support. However, some may shape the neuropathic phenotype through mechanisms other than impaired cargo movement.

Vitamin B12 deficiency represents an additional clinically relevant and modifiable contributor that should be considered in patients with diabetes and neuropathy. This issue is particularly important in patients treated with metformin, because long-term metformin use has been associated with reduced vitamin B12 concentrations and biochemical vitamin B12 deficiency [124,125]. It may also be relevant in patients receiving long-term treatment with drugs that reduce gastric acid secretion, such as proton pump inhibitors or histamine-2 receptor antagonists, for coexisting gastrointestinal disease, because these drugs have been associated with reduced vitamin B12 status [126]. Vitamin B12 deficiency may cause or worsen peripheral neuropathy and may overlap clinically with diabetic neuropathy, especially when sensory loss, paresthesia, gait imbalance, or autonomic symptoms are present [127]. In the context of the present review, vitamin B12 deficiency should be interpreted primarily as a potentially independent or aggravating risk factor, and not as an established direct mechanism of diabetic axonal transport failure [124,125,127]. It may, however, influence axonal maintenance indirectly through altered methylation and homocysteine metabolism, increased homocysteine, and impaired neuronal or glial support [124,127,128]. Assessment of vitamin B12 status is thus important in clinical studies of DN, particularly when evaluating neuropathy progression in patients exposed to long-term metformin treatment, prolonged use of proton pump inhibitors, or other drugs that reduce gastric acid secretion [124,125,126,127].

## 9. Conclusions

Diabetic neuropathy can be viewed, in part, as an axonal-maintenance disorder. Metabolic, cellular, and cytoskeletal mechanisms place pressure on long peripheral axons. The most direct experimental evidence shows impaired slow transport of cytoskeletal proteins, reduced neurofilament and tubulin synthesis and export, altered retrograde transport, reduced axonal caliber, and defective regeneration. These changes provide a coherent cellular explanation for distal fiber loss, sensory deficits, pain, impaired conduction, and selected autonomic manifestations. At the same time, evidence related to carbonyl stress, mitochondrial or vascular dysfunction, neuroimmune activation, and Schwann cell injury should be interpreted mainly as mechanistic support unless axonal transport was directly measured.

RAGE–DIAPH1 signaling may provide a plausible link between AGE-rich tissue and cytoskeletal remodeling, particularly actin regulation. It should, however, be presented as one component of a larger process involving axons, glia, and vascular structures, and not as the sole cause of DN. Future research should connect biochemical stress, receptor signaling, mitochondrial trafficking, motor behavior, and cytoskeletal cargo movement within a single experimental system. Translational studies should prioritize structural and functional endpoints that reflect axonal integrity. This distinction is necessary to determine which pathways directly impair axonal transport and which mainly shape the axon–glia–vascular environment of DN.

## Figures and Tables

**Table 1:** Mechanistic links between diabetic stress signals and axonal maintenance failure.

Model/Material	Mechanistic Route and Principal Target	Axonal Maintenance Association	Evidence Category
Human diabetic sural nerve [12]	Glycation of structural proteins: cytoskeletal and myelin proteins	Direct chemical modification of structural substrates	Direct structural substrate modification
Experimental diabetes [13]	Glycation of nerve proteins: sciatic nerve cytoskeletal proteins	Peripheral nerve cytoskeleton is a target of chronic glycation	Direct structural substrate modification
AGE-modified extracellular matrix in vitro [14]	Matrix glycation: sensory neurite outgrowth	Glycated matrix limits regenerative growth	Transport-relevant but indirect
Primary sensory neurons [15]	RAGE-induced oxidative stress: DRG neurons	RAGE activation injures neurons through ROS and apoptosis	Transport-relevant but indirect
RAGE ligand-stimulated cells [22]	RAGE–DIAPH1 coupling: DIAPH1, Rac1, and Cdc42	Connects RAGE ligands with actin regulatory machinery	Mechanistic inference
Structural RAGE-mDia1 studies [23]	Receptor–effector interaction: RAGE cytoplasmic tail and mDia1	Defines the molecular interface for RAGE-dependent signaling	Mechanistic inference
Cell models of mDia1 activity [62]	Actin–microtubule coordination: F-actin and microtubules	Provides a route by which DIAPH1 may affect transport tracks	Mechanistic inference
Axonal cytoskeleton imaging [19]	Periodic axonal scaffold: actin, spectrin, and adducin	Defines structural organization required for axonal stability	Transport-relevant but indirect
Axonal cytoskeleton disruption [20]	Actin-dependent microtubule stability: axonal microtubules	Actin disruption destabilizes microtubule tracks	Mechanistic inference
Diabetic DRG neurons [11,70]	Mitochondrial respiration/AMPK signaling: DRG neurons	Reduced energy supply may weaken cargo movement and growth	Transport-relevant but indirect
Schwann cells exposed to carbonyl or AGE stress [46,50]	Schwann cell injury: Schwann cells	Loss of glial support can indirectly compromise axonal transport	Glial support mechanism
Human DRG and diabetic mice [16,17]	Neuroimmune activation: DRG neurons and macrophages	Inflammation alters the soma and nerve environment for transport	Clinical association; direct retrograde transport evidence

Summary of experimental and translational evidence linking glycation, AGE–RAGE signaling, RAGE–DIAPH1 coupling, mitochondrial dysfunction, Schwann cell injury, neuroimmune activation, and cytoskeletal instability with impaired axonal maintenance in diabetic neuropathy.

**Table 2:** Experimental evidence of axonal transport and cytoskeletal maintenance defects in diabetic neuropathy.

Model/Material	Transport Domain	Main Finding	Interpretation
STZ diabetic rats after sciatic nerve crush [95]	Retrograde transport	Altered accumulation of labeled proteins and glycoproteins	Early evidence of impaired axon-to-soma communication
Mutant diabetic mice [4]	Slow cytoskeletal transport	Altered transport of actin, tubulin, and neurofilament proteins	Structural cargo delivery is disturbed
STZ diabetic rats [94]	Metabolic enzyme transport	Impaired phosphofructokinase transport	Transport failure is not limited to cytoskeletal proteins
Experimental diabetes models [3]	Slow axonal transport	Reduced transport of neurofilaments, tubulin, actin, and glycolytic enzymes	Broad impairment of slow cargo movement
STZ diabetic rats [5]	Slow cytoskeletal transport	Impaired transport of cytoskeletal proteins in the sciatic nerve	Direct peripheral nerve evidence
Chronic diabetic rats [6]	Axonal caliber	Reduced fiber size correlated with neurofilament loss	Connects cytoskeletal cargo loss with axonal atrophy
STZ diabetic rats [7]	DRG synthesis and distal export	Reduced neurofilament and α-tubulin mRNA; distal loss of cytoskeletal proteins	Cargo production and delivery are both affected
STZ diabetic rats [96]	Stress signal transport	Increased retrograde transport of phosphorylated JNK and p38	Diabetes alters selected retrograde signaling cargoes
Type 1 diabetic BB/Wor rats after sciatic nerve crush [8]	Regenerative transport response	Blunted DRG tubulin response with impaired axonal elongation and caliber growth	Regeneration reveals defective cytoskeletal remodeling in diabetic sensory neurons
Diabetic mice [17]	Retrograde transport	Macrophage RAGE slowed retrograde transport and reduced neuronal insulin sensitivity	Neuroimmune signaling can impair axon-to-soma maintenance

Summary of experimental studies linking diabetes with altered retrograde transport, slow cytoskeletal cargo movement, metabolic enzyme transport, regenerative cytoskeletal responses, and axon-to-soma communication.

**Table 3:** Therapeutic strategies and translational readouts relevant to axonal preservation in diabetic neuropathy.

Model/Material	Strategy or Readout	Level	Endpoint	Main Value
Impaired glucose tolerance with neuropathy [114]	Diet and exercise	Metabolic/lifestyle	IENFD, pain	Shows structural small-fiber improvement
Diabetes without neuropathy [115]	Supervised exercise	Metabolic/lifestyle	Cutaneous nerve density	Suggests early distal axon preservation
Diabetic mice [30]	Glyoxalase-1 elevation	Carbonyl detoxification	IENFD, DRG OXPHOS proteins	Links dicarbonyl clearance with fiber preservation
Experimental diabetic neuropathy [116]	Aminoguanidine	AGE/carbonyl inhibition	MNCV, nerve structure	Preclinical support for anti-glycation treatment
Sensory neurons and diabetic mice [15,17]	RAGE blockade	Neuronal and immune signaling	ROS, DRG size, retrograde transport	Plausible target for preserving axon-to-soma communication
Cellular and diabetic models [65,66,67]	RAGE-DIAPH1 inhibition	Cytoskeletal signaling	RAGE-DIAPH1 interaction, inflammation	Mechanistically attractive, neuropathy data remain limited
Clinical diabetic neuropathy trials [117,118]	α-lipoic acid	Oxidative stress	Symptoms, deficits, composite endpoints	Functional benefit with limited transport specificity
Chronic diabetic neuropathy trials [119]	Acetyl-L-carnitine	Mitochondrial/regenerative support	Pain, nerve fiber regeneration, vibration perception	Compatible with an axonal preservation model
Type 1 diabetes trial [120]	Omega-3 fatty acids	Small-fiber support	Corneal nerve fiber length	Noninvasive structural regeneration signal
Clinical diabetic neuropathy cohorts [107,108]	Corneal confocal microscopy	Small-fiber readout	CNFL and related measures	Tracks small sensory axon integrity
Clinical diabetic neuropathy cohorts [101,102,109]	Skin biopsy and sudomotor testing	Distal small-fiber/autonomic readouts	IENFD, ESC	Captures distal structural and autonomic involvement

Summary of interventions and readouts relevant to distal axonal preservation in diabetic neuropathy, including lifestyle interventions, carbonyl detoxification, anti-glycation approaches, RAGE and RAGE–DIAPH1 signaling, antioxidant and mitochondrial support, corneal nerve assessment, skin biopsy, sudomotor testing, and nerve conduction measures.

## Data Availability

No new data were created or analyzed in this study. Data sharing is not applicable to this article.

## Abbreviations

The following abbreviations are used in this manuscript:

AGEs	Advanced glycation end products
ALA	α-Lipoic acid
AMPK	Adenosine monophosphate-activated protein kinase
AUC	Area under the curve
BB/Wor	BioBreeding/Worcester
CML	N-ε-carboxymethyllysine
CNFL	Corneal nerve fiber length
DIAPH1	Diaphanous-related formin 1
DN	Diabetic neuropathy
DPN	Diabetic peripheral neuropathy
DRG	Dorsal root ganglion/ganglia
DRP1	Dynamin-related protein 1
ESC	Electrochemical skin conductance
GFAP	Glial fibrillary acidic protein
GO	Glyoxal
HMGB1	High mobility group box 1
IB4	Isolectin B4
IENFD	Intraepidermal nerve fiber density
IL-6	Interleukin-6
ISR	Integrated stress response
MG-H1	Methylglyoxal-derived hydroimidazolone 1
MGO	Methylglyoxal
MNCV	Motor nerve conduction velocity
MRP1	Multidrug resistance-associated protein 1
NAC	N-acetyl-L-cysteine
NF-κB	Nuclear factor kappa-light-chain-enhancer of activated B cells
NLRP3	NLR family pyrin domain-containing protein 3
Nrf2	Nuclear factor erythroid 2-related factor 2
OXPHOS	Oxidative phosphorylation
PARP	Poly(ADP-ribose) polymerase
PGC-1α	Peroxisome proliferator-activated receptor gamma coactivator 1-alpha
PMP22	Peripheral myelin protein 22
RAGE	Receptor for advanced glycation end products
ROS	Reactive oxygen species
SN	Sciatic nerve
STZ	Streptozotocin
T1DM	Type 1 diabetes mellitus
T2DM	Type 2 diabetes mellitus
TGF-β	Transforming growth factor beta
TIMP-1	Tissue inhibitor of metalloproteinases 1
TLR4	Toll-like receptor 4
TNF-α	Tumor necrosis factor alpha
TRPA1	Transient receptor potential ankyrin 1
TRPM8	Transient receptor potential cation channel subfamily M (melastatin) member 8
VEGF	Vascular endothelial growth factor
3-DG	3-Deoxyglucosone
